# Interaction of CYP3A4 with caffeine: First insights into multiple substrate binding

**DOI:** 10.1016/j.jbc.2023.105117

**Published:** 2023-07-29

**Authors:** Irina F. Sevrioukova

**Affiliations:** Department of Molecular Biology and Biochemistry, University of California, Irvine, California, USA

**Keywords:** cytochrome P450, CYP3A4, ligand-binding protein, caffeine, complex, crystal structure, spectroscopy

## Abstract

Human cytochrome P450 3A4 (CYP3A4) is a major drug-metabolizing enzyme that shows extreme substrate promiscuity. Moreover, its large and malleable active site can simultaneously accommodate several substrate molecules of the same or different nature, which may lead to cooperative binding and allosteric behavior. Due to difficulty of crystallization of CYP3A4-substrate complexes, it remains unknown how multiple substrates can arrange in the active site. We determined crystal structures of CYP3A4 bound to three and six molecules of caffeine, a psychoactive alkaloid serving as a substrate and modulator of CYP3A4. In the ternary complex, one caffeine binds to the active site suitably for C8-hydroxylation, most preferable for CYP3A4. In the senary complex, three caffeine molecules stack parallel to the heme with the proximal ligand poised for 3-N-demethylation. However, the caffeine stack forms extensive hydrophobic interactions that could preclude product dissociation and multiple turnovers. In both complexes, caffeine is also bound in the substrate channel and on the outer surface known as a peripheral site. At all sites, aromatic stacking with the caffeine ring(s) is likely a dominant interaction, while direct and water-mediated polar contacts provide additional stabilization for the substrate-bound complexes. Protein-ligand interactions *via* the active site R212, intrachannel T224, and peripheral F219 were experimentally confirmed, and the latter two residues were identified as important for caffeine association. Collectively, the structural, spectral, and mutagenesis data provide valuable insights on the ligand binding mechanism and help better understand how purine-based pharmaceuticals and other aromatic compounds could interact with CYP3A4 and mediate drug-drug interactions.

Caffeine (1,3,7-trimethylxanthine; Fig. 1) is the most widely consumed psychoactive alkaloid. The popularity of caffeine is related to its stimulant properties on the central nervous system, manifested as elevated mood, decreased fatigue, and increased capacity for work. Caffeine is naturally found in seeds, leaves, and fruits of many plants and can be manufactured through chemical synthesis. The largest contributors to dietary caffeine are coffee, tea, and caffeinated soft drinks.

**Figure 1:**
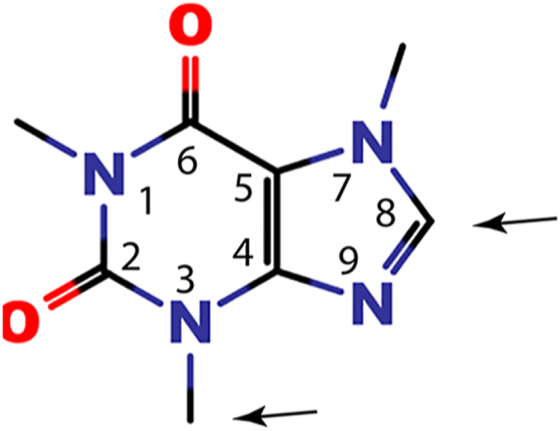
**Chemical structure of caffeine**. *Arrows* indicate sites of metabolism catalyzed by CYP3A4: C8 atom and 3-N-methyl. CYP3A4, cytochrome P450 3A4.

Caffeine is quickly absorbed in the stomach and small intestines and acts through multiple mechanisms but primarily by blocking adenosine receptors. Daily consumption of up to 400 mg of caffeine (equivalent to four cups of brewed coffee, ten cans of cola, or two caffeinated drinks) is considered safe for adults (1). The maximal plasma concentration (C_max_) up to 10 mg/L (∼50 μM) is generally safe, whereas at excessive doses (C_max_ of 30–50 mg/L or 150–250 μM) caffeine may exert toxicity and cause psychotic effects (2). Caffeine is also used for therapeutic purposes, mainly as a component of combination prescription drugs and over-the-counter medications. Consumption of high levels of caffeine may result in pharmacokinetic interactions with other medications (2) or lead to the development of tolerance to its pharmacologic effects.

One mechanism of drug-drug interactions is formation of π-π stacking complexes between caffeine and polyaromatic pharmaceuticals, such as daunomycin, doxorubicin, and mitoxantrone, which could decrease their effective concentration (3). Through formation of mixed π-π complexes, caffeine can also sequester other biologically active compounds, for example, neurotoxin 1-methyl-4-phenyl-1,2,3,6-tetrahydropyridine (4) and mutagenic/carcinogenic heterocyclic aromatic amines (5). In the aqueous environment, caffeine undergoes self-association, forming dimers and higher order clusters (6, 7). The tendency to self-oligomerization is thought to be physiologically important, as it could contribute to anomalous responses of caffeine in the central nervous system (8).

Another mechanism of caffeine-drug interactions is direct binding of caffeine to the active site of drug-metabolizing enzymes and modulation of their activity (2, 9). Caffeine metabolism is complex and involves multiple enzymes and products, with over 25 metabolites identified in humans so far (10). Demethylation is the major route of caffeine metabolism (>90%). Among three possible demethylation reactions, 3-N-demethylation accounts for 84% and results in paraxanthine formation. CYP1A2 mediates over 95% of the primary caffeine metabolism including 3-N-demethylation (11). Other caffeine metabolizing enzymes are CYP2E1, CYP2D6, and CYP3A4, which may act as low-affinity, high-capacity isoforms catalyzing N-demethylation and 8-hydroxylation. The latter reaction leads to formation of 1,3,7-trimethyl uric acid and is predominantly catalyzed by CYP3A4 (12). Despite the high *K*_m_ and low *V*_max_ values (0.91 mM and 0.157 min^−1^, respectively, in Supersomes (12)), CYP3A4 could significantly contribute to caffeine metabolism because it is the most abundant CYP in the liver.

Whether caffeine can interfere with the CYP3A4-dependent xenobiotic metabolism and mediate drug-drug interactions *in vivo* is yet unknown. However, it was reported that caffeine has multiple binding sites in recombinant CYP3A4 and modulates its enzymatic activity (9). Therefore, it was of interest to investigate in more detail how CYP3A4 interacts with caffeine to better understand the ligand binding and modulatory mechanism. Here, we present crystal structures of Δ3-22 CYP3A4 bound to three and six caffeine molecules, as well as experimental results, that provide the first insights into the multiple substrate binding, emphasize the importance of remote ligand docking areas, and identify key residues interacting with caffeine.

## Results and discussion

### Caffeine is a low-affinity type I ligand

Like all other P450 enzymes, CYP3A4 contains the heme cofactor, whose absorption wavelength (λ_max_) and amplitude depend on the redox and coordination state of the heme iron. In the resting state, ferric CYP3A4 is in a low-spin six-coordinated state absorbing at ∼416 nm, with water serving as the displaceable distal ligand. Upon substrate binding and dissociation of the water ligand, the Soret band shifts to the 385-395 nm region (blue shift or type I spectral change), manifesting a low-to-high spin transition in the heme iron. Depending on the substrate affinity and spatial fit, this spectral shift can be partial or complete.

**Figure 2:**
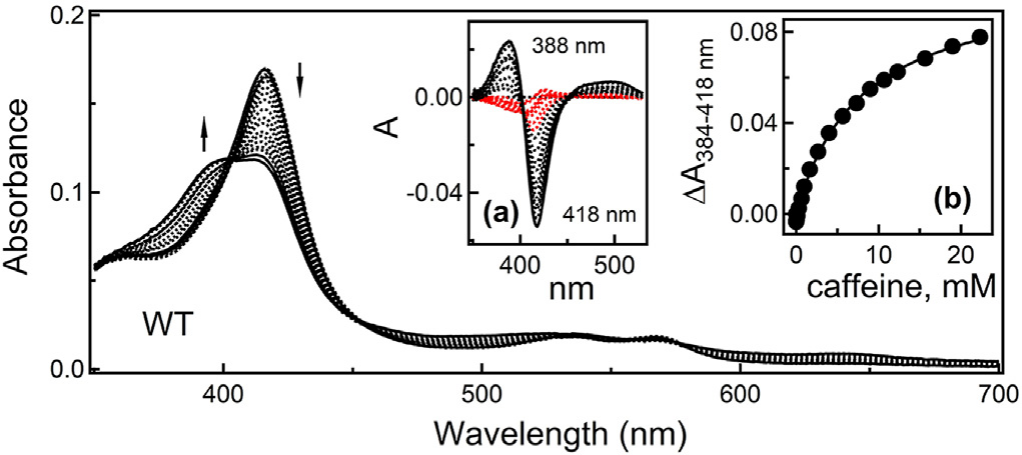
**Spectral changes observed upon binding of caffeine to Δ3-22 CYP3A4**. *Arrows* indicate direction of absorbance changes. Difference spectra are shown in inset *A*. Absorbance changes observed during the first spectral phase, characterized by a decrease in the Soret band with no rise in the 360−400 nm region, are highlighted in *red*. Inset *B* is a titration plot with sigmoidal fitting. The derived parameters are listed in Table 1. CYP3A4, cytochrome P450 3A4.

Spectral titrations showed that caffeine acts as a type I ligand for Δ3-22 CYP3A4, lacking the N-terminal trans-membrane helix (Fig. 2). However, based on the difference spectra (inset *a*), two spectral phases were distinguished. The first phase was observed at caffeine concentrations < 1 mM (red spectra in inset *a*) and characterized by a decrease in the Soret band with no increase in the 360-400 nm region. At higher caffeine concentrations, there was a further decrease in the Soret band accompanied by a rise in the 360 to 400 nm absorbance, indicative of a low-to-high spin transition in the heme iron. The spin conversion was partial, with the high-spin content reaching 48% at the end of the experiment. The titration plot (inset *b*) could be equally well fitted with the hyperbolic or Hill equations, giving the *K*_d_ or *S*_50_ values of ∼8 mM and the Hill coefficient of 1.0 (Table 1). This implies that, within the studied concentration range, only one ligand binds near the heme and alters its environment.

**Table 1:** Binding parameters for the interaction of caffeine with WT and mutants of CYP3A4

CYP3A4	S_50_^a^ mM	n_H_^b^	K_d_^c^ mM	High spin %
WT	7.8 ± 0.2	1.0 ± 0.0		48 ± 1
R212A	8.9 ± 0.2	0.91 ± 0.01		46 ± 2
T224A	30 ± 5	0.50 ± 0.01		44 ± 3
F219A			0.18 ± 0.05 d (10%) e 15 ± 3 f	41 ± 2

All values are the average of three independent measurements with standard deviation.

a Caffeine concentration at half-saturation (equivalent to K_d_).

b Hill coefficient.

c Spectral dissociation constant.

d Value for the high-affinity site.

e Percentage of the absorbance change due to association of caffeine to the high-affinity site.

f Value for the low-affinity site.

In contrast, two independent caffeine-binding sites with *K*_d_ of 62 μM and 12.9 mM were detected in full-length CYP3A4 (9). To understand the origin of this discrepancy, we repeated the titration experiment with full-length CYP3A4 (Fig. S1) and found that, indeed, the titration curve was best fitted with the 2-site binding model, giving comparable *K*_d_ values for the high- and low-affinity sites: 20.5 ± 0.6 μM and 4.5 ± 0.3 mM, respectively. The distinct behavior of two protein forms was not surprising because the N-terminal helix is known to promote CYP3A4 aggregation that alters interaction with substrates (13). Even so, the percentage of the absorbance change due to the association of caffeine to the high-affinity site in full-length CYP3A4 was low (9%), meaning that the low-affinity site remains the preferable docking area. Although full-length CYP3A4 is a biologically relevant protein form, the catalytically active Δ3-22 CYP3A4 was used in this study to better relate spectral and structural results.

### Crystallization of CYP3A4-caffeine complexes

During crystallization trials, two caffeine concentrations were used: 22 mM, the maximal concentration in spectral experiments, and 80 mM to achieve better ligand saturation. In the latter case, the protein was mixed with a preheated solution of 250 mM caffeine. Well-diffracting crystals grew under both conditions and in the same space group that contained one molecule per asymmetric unit. Structures were determined to 2.05 to 2.15 Å resolution (Table 2) and, overall, were highly similar, with the r.m.s. deviation between the C_α_ atoms of 0.237 Å. The main difference was in the number and association mode of caffeine molecules bound to CYP3A4.

**Table 2:** Data collection and refinement statistics

CYP3A4-caffeine complex		
PDB ID	8SO1	8SO2
Data statistics		
Space group	I222	I222
Unit cell parameters	*a* = 78 Å, *b* = 101 Å, *c* = 131 Å α, β, γ = 90°	*a* = 78 Å, *b* = 101 Å, *c* = 133 Å α, β, γ = 90°
Resolution range (Å)	79.93–2.05 (2.16–2.05) a	80.12–2.15 (2.27–2.15)
Total reflections	154,383	132,953
Unique reflections	32,412 (4693)	28,315 (4096)
Redundancy	4.8 (4.8)	4.7 (4.9)
Completeness	99.2 (99.4)	98.9 (99.5)
Average *I*/*σI*	8.3 (1.0)	6.6 (1.0)
R_merge_	0.060 (1.690)	0.093 (1.784)
R_pim_	0.030 (0.841)	0.046 (0.871)
CC ½	0.997 (0.451)	0.996 (0.371)
Refinement statistics		
*R*/*R*_free_^b^	20.5/23.8	20.9/25.3
Number of atoms:		
Protein	3772	3785
Solvent	79	121
r.m.s. deviations:		
Bond lengths, Å	0.008	0.003
Bond angles, °	0.958	0.593
Wilson B-factor, Å^2^	62	57
Average *B*-factor, Å^2^:		
Protein	89	79
Caffeine ligands c	71, 117, 69	64, 76, 73; 81; 62, 70
Ramachandran plot d (residues; %)		
Preferred	443 (95.1%)	448 (95.7%)
Allowed	23 (4.9%)	20 (4.3%)
Outliers	0	0

a Values in brackets are for the highest resolution shell.

b *R*_free_ was calculated from a subset of 5% of the data that were excluded during refinement.

c Values correspond to ligands bound to the active site, substrate channel, and peripheral site, respectively.

d Analyzed with PROCHECK.

### Crystal structure of CYP3A4 bound to three caffeine molecules

**Figure 3:**
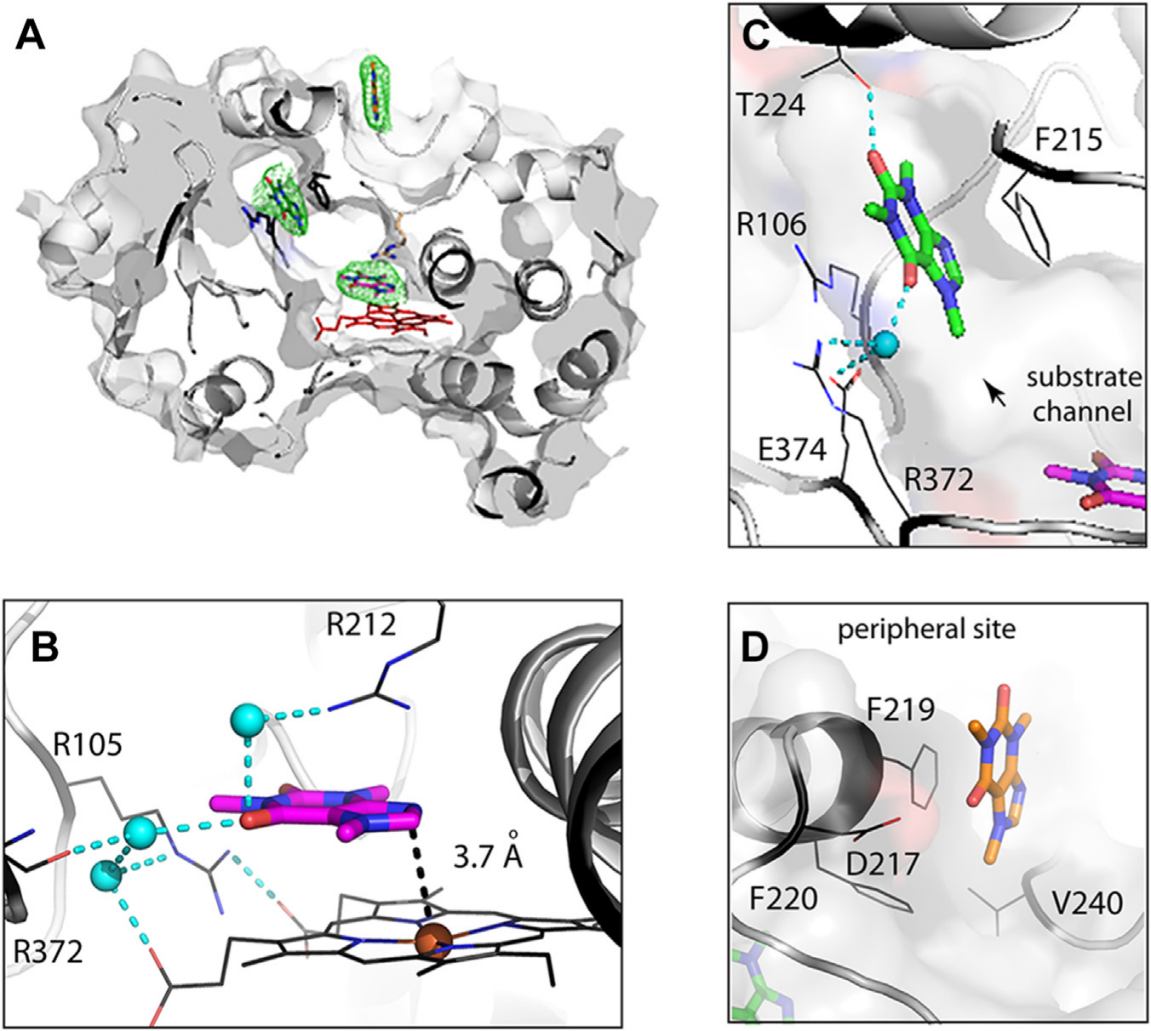
**Crystal structure of Δ3-22 CYP3A4 bound to three caffeine molecules (ternary complex; PDB ID****8SO1****).***A*, slice through the CYP3A4 molecule showing location of caffeines. *Greenmesh* is a polder omit electron density map contoured at 3σ level. Close-up views at the electron density maps for individual caffeine ligands are shown in Fig. S2. *B*, caffeine bound to the active site near the catalytic center. The ligand orientation is productive (Fe-C8 distance < 4.0 Å) and stabilized through aromatic stacking with the heme, cation-π interactions with the R212 guanidine, and water-mediated hydrogen bonds connecting the carbonyl oxygen of caffeine to the heme propionates and arginines 105, 212, and 372. *C*, the second caffeine molecule binds in the substrate channel between/parallel to the F215 ring and the R106 guanidine, forming π-π and cation-π interactions, respectively. In addition, this caffeine is directly H-bonded to T224 and, *via* the water molecule, to R372 and E374. *D*, the third caffeine docks at the peripheral site parallel to the F219 ring. The D217 carboxyl group is 3.5 Å away from the carbonyl oxygen of caffeine, allowing a long-range polar interaction. In panels *B* and *C*, water molecules and H-bonds are shown as *cyan balls* and *cyan dashed lines*, respectively. CYP3A4, cytochrome P450 3A4; PDB, Protein Data Bank.

The structure obtained at lower caffeine concentration contained three caffeine molecules: one in the active site, one in the substrate channel, and one bound on the outer surface (Fig. 3*A*). In this structure, named “ternary complex”, the active site caffeine stacks parallel to the heme, with the C03 atom (C8 in Fig. 1) being 3.7 Å away from the catalytic center (Fig. 3*B*). Thus, caffeine is in a productive mode and positions suitably for oxidation at the most preferable position (12). Besides aromatic stacking with the heme cofactor, the caffeine orientation is stabilized by cation-π interactions with the R212 guanidine group and water-mediated H-bonds connecting the carbonyl oxygen (O6 in Fig. 1) to the heme propionates and the main/side chains of R105, R212, and R372.

The second caffeine molecule stacks in the substrate channel between and parallel to the F215 and R106 side chains, forming π-π and cation-π interactions, respectively (Fig. 3*C*). In addition, the intrachannel caffeine establishes direct and water-mediated H-bonds with T224, R372, and E374. The third ligand docks on the outer surface in a hydrophobic grove known as a peripheral site (14). Here, caffeine stacks parallel to the F219 ring (∼3.8 Å apart; Fig. 3*D*) and forms van der Waals contacts with F220, I238, C239, and V240, and a long-range polar interaction with the D217 carboxyl (3.5 Å away). At all three sites, aromatic stacking is likely a major contributor to the caffeine binding.

Because caffeine concentrations used for titration experiments and crystallization of the ternary complex were within the same range, structural findings can be related to spectral data. Equilibrium titrations inform only on ligand-dependent changes in the heme coordination and, as spectral data suggest (Fig. 2 and Table 1), a single caffeine molecule alters the heme environment. Out of three ligands seen in the crystal structure, the active-site caffeine is likely responsible for that. The delay in the high-spin transition could be explained by an existence of two orientations of the active-site caffeine. The initial orientation, adopted at low caffeine concentrations (≤1 mM), would be suboptimal or too far from the heme to induce spin shift. At higher ligand concentrations, the remote site(s) would be occupied and, through a conformational change, could force the active-site caffeine to reorient or shift closer to the heme, thereby promoting spin transition. The existence of an alternative orientation of the active-site caffeine could explain its slow oxidation under physiological conditions (≤100 μM) (2). Concordantly, studies on CYP3A4-dependent metabolism of aliphatic derivatives of theobromine, a caffeine analogue lacking the N1-methyl, suggested that the theobromine moiety binds within the four-carbon-atom distance from the heme iron (15). The same area may serve as the initial docking site for caffeine as well.

Another explanation for the delay in the spin transition would be the association of caffeine to a remote area that has the same affinity as the active site. Because equilibrium titrations do not report on the ligands’ binding area and orientation, it was not possible to differentiate, based solely on spectral data, the occupation of which site, the intrachannel or peripheral one, could induce absorbance changes or trigger repositioning of the ligand bound to the active site. However, as discussed below, site-directed mutagenesis helped to clarify this matter.

### Crystal structure of CYP3A4 bound to six caffeine molecules

**Figure 4:**
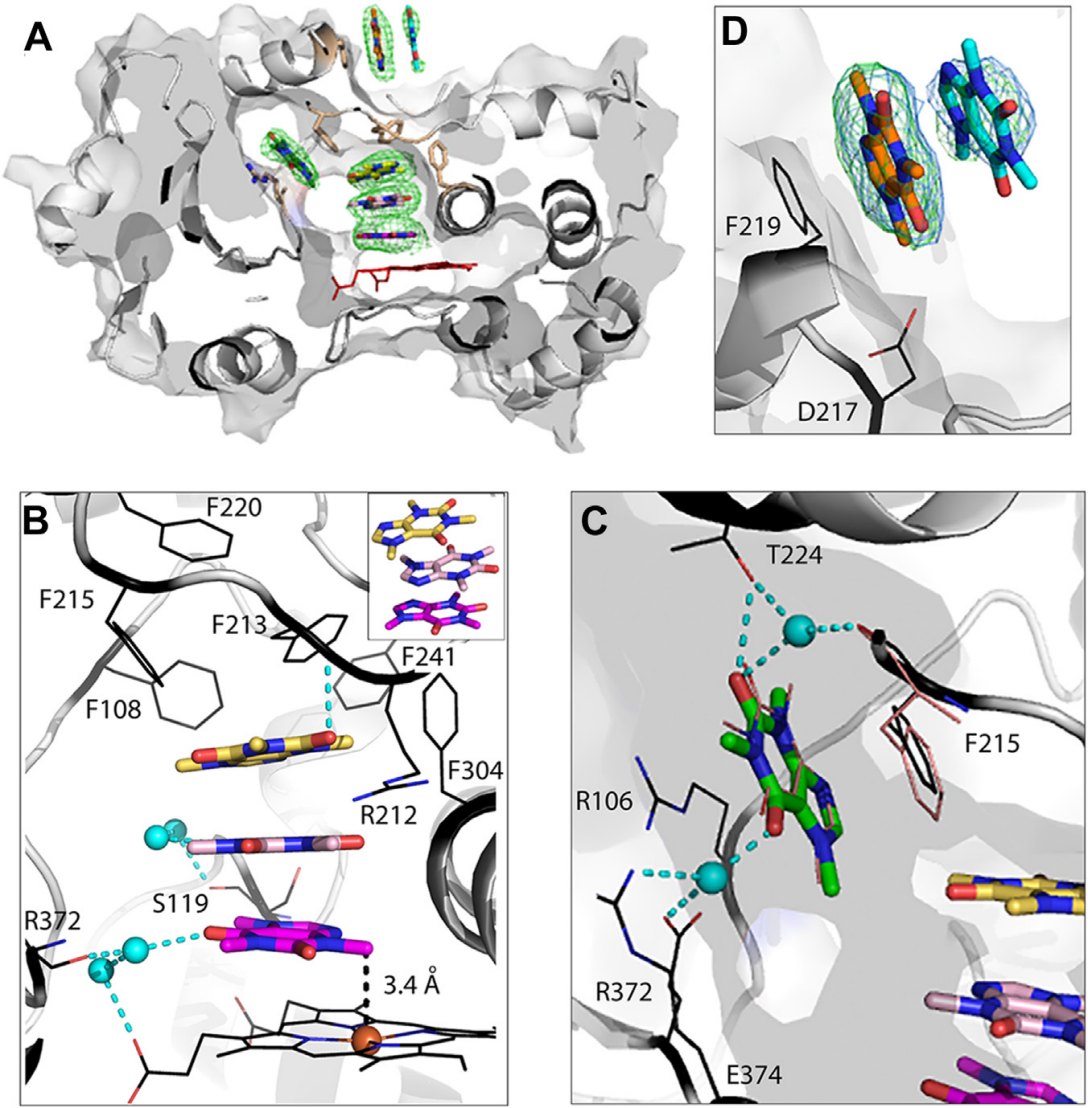
**Crystal structure of Δ3-22 CYP3A4 bound to six caffeine molecules (senary complex; PDB ID****8SO2****).***A*, slice through the CYP3A4 molecule showing location of caffeines. *Greenmesh* is a polder omit electron density map contoured at 3σ level. Close-up views at the electron density maps for individual caffeine ligands are shown in Fig. S2. *B*, stack of three active-site caffeine molecules bound on top of the heme. The proximal ligand is in a productive orientation suitable for 3-N-demethylation. The complex is stabilized through parallel π-π stacking and water-mediated H-bonds connecting caffeine ligands to the main chain atoms of S119, F213, and R372 and to the heme propionate. The upper caffeine forms extensive aromatic interactions with phenylalanines 108, 213, 215, 241, and 304. Inset is another view at the caffeine trimer showing alternate monomer orientations. *C*, the fourth caffeine stacks in the substrate channel between/parallel to the F215 ring and the R106 guanidine, and establishes H-bonded water bridges with T224, R372, and E374. For comparison, the intrachannel caffeine and F215 in the ternary complex are shown in *pink lines*. *D*, two more caffeine monomers bind to the peripheral site by stacking parallel to the F219 ring. *Blue* and *greenmesh* are 2F_o_- F_c_ and omit polder maps contoured at 1σ and 3σ level, respectively. The D217 carboxyl is close enough to the carbonyl oxygen of the proximal caffeine (3.5 Å away) to mediate a long-range polar interaction. In panels *B* and *C*, *cyan balls* and *cyan dotted lines* represent water molecules and H-bonds, respectively. CYP3A4, cytochrome P450 3A4; PDB, Protein Data Bank.

At higher caffeine concentration in crystallization solution, six ligands cocrystallized with CYP3A4. In the structure, termed “senary complex”, a stack of three caffeines occupies the active site, one monomer docks in the substrate channel and two more at the peripheral site (Fig. 4*A*). The active site caffeines lay on top of each other in alternating orientations and parallel to the heme (Fig. 4*B*). A strong π-π overlap is evident from short intermolecular distances (3.3 Å, on average). In stacks of caffeine, the antiparallel alignment geometry minimizes steric clashing between the methyl groups and optimizes dipolar interactions (16, 17). To accommodate the caffeine trimer, R212 swings aside and F304 adopts a vertical rotamer. The caffeine monomers connect to the main chain atoms of S119, F213, or R372 *via* direct or water-mediated H-bonds. The lower caffeine is also linked *via* a water bridge to the heme propionate. Compared to the active-site caffeine in the ternary complex (Fig. 3*B*), the lower monomer orients distinctly, with the 3-N-methyl being the closest to the heme iron (3.4 Å away). Again, this is a productive orientation for an alternative oxidation site. *In vivo*, this binding mode is less favorable, because the 3-N-demethylation of caffeine is predominantly catalyzed by CYP1A2 and only negligibly by CYP3A4 (12). The upper caffeine is surrounded by five phenylalanine residues (F108, F213, F215, F241, and F304) from the Phe-cluster, present uniquely in CYP3A4 (18). Such an arrangement results in extensive hydrophobic interactions that might be hard to break. If true, then multiple turnovers in the senary complex would not be possible because, even if the lower caffeine undergoes oxidation, the product will be unable to dissociate. Thus, the senary complex might be nonfunctional. Considering the high tendency of caffeine to self-aggregation, particularly in oversaturated solutions, it is likely that a preformed trimer rather than three monomers of caffeine consecutively entered the active site. In either case, a stack of caffeines crowding and clogging the catalytic cavity provides a glimpse on how small aromatic molecules can arrange to inhibit CYP3A4 activity (19) and mediate drug-drug interactions (9).

As observed in the ternary complex, the intrachannel caffeine stacks between the F215 ring and the R106 guanidine and forms direct and water-mediated H-bonds with the side chains of T224, R372, and E374 (Fig. 4*C*). Two main distinctions are an additional water bridge with the F215 carbonyl and an upward shift of the F215 side chain. The latter movement minimizes steric clashing with the caffeine trimer and improves aromatic overlap with the caffeine ring. As a result, the intrachannel caffeine has lower thermal motion and is better defined than in the ternary complex.

At the peripheral site, two caffeine monomers stack parallel to each other and the F219 ring, with an average interring distance of ∼3.6 Å (Fig. 4*D*). The proximal ligand (closest to F219) is well-defined and orients similarly to that in the ternary complex. The distal caffeine, however, is only partially defined, possibly due to a lower occupancy: 70% *versus* 97% for the proximal site.

### Role of the active site R212

**Figure 5:**
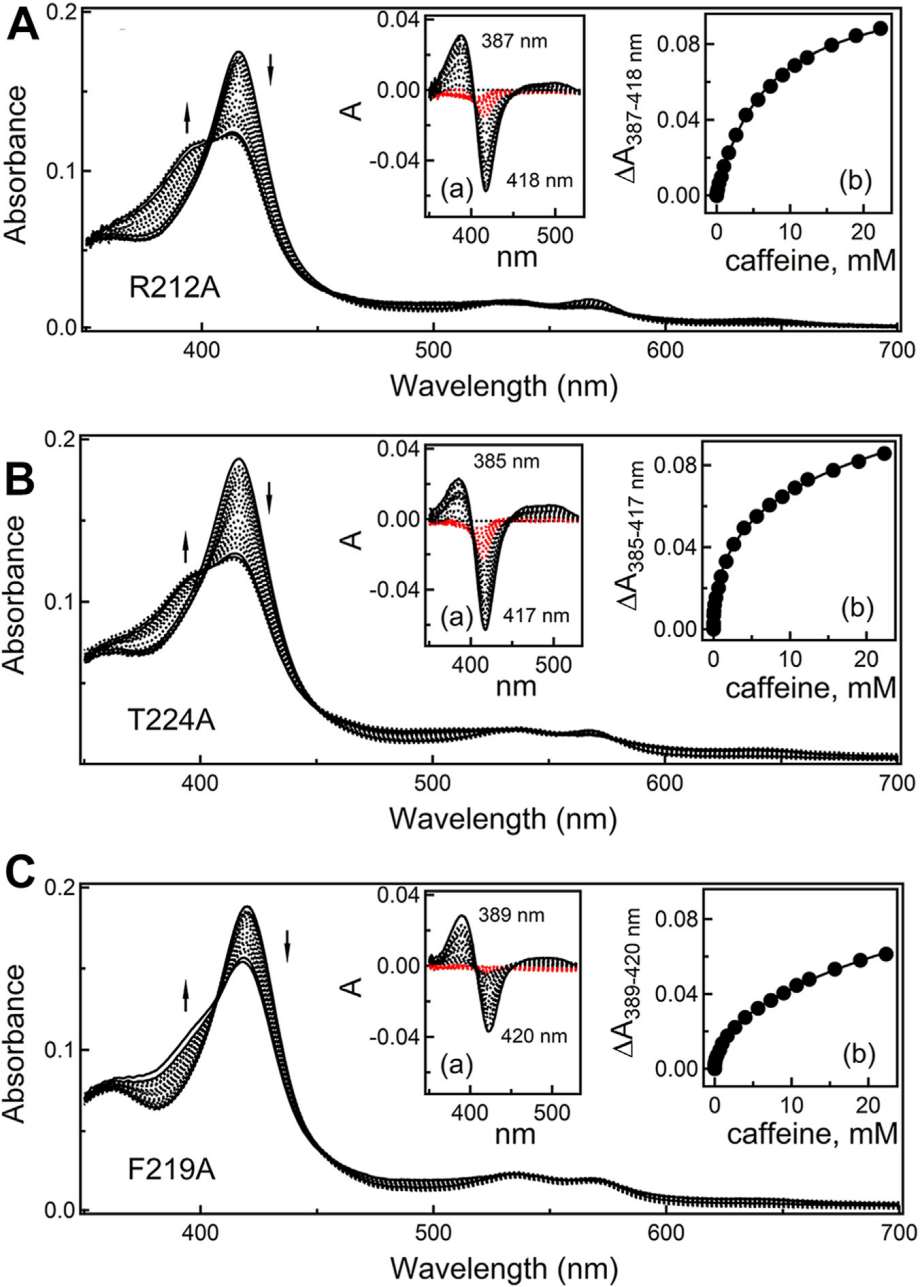
**Spectral changes induced by caffeine in CYP3A4 mutants**. *A*–*C*, spectral changes induced by caffeine in R212A, T224A and F219A mutants of Δ3-22 CYP3A4, respectively. *Arrows* indicate direction of absorbance changes. Insets *a* show difference spectra, where absorbance changes occurring during the first spectral phase, characterized by a decrease in the Soret band with no rise in the 360−400 nm region, are highlighted in *red*. Insets *b* display titration plots with sigmoidal or hyperbolic fittings. The derived parameters are listed in Table 1. CYP3A4, cytochrome P450 3A4

Crystal structures suggest that R212 could have a dual effect on the caffeine binding. In the ternary complex, R212 helps to orient the active-site caffeine and strengthens the complex through cation-π and H-bonding interactions mediated by the guanidine group (Fig. 3*B*). In the senary complex, the bulky side chain of R212 interferes and moves away to allow the caffeine trimer to approach the heme (Fig. 4*B*). However, due to space limitations, it cannot move far enough and clashes with the top monomer (3.0 Å away), causing distortion of the caffeine stack. So, it was of interest to investigate whether elimination of the R212 side chain affects the complex formation with caffeine. The R212A mutant was already available and previously used for analyzing CYP3A4-ligand interactions (20, 21, 22). According to the spectral data (Fig. 5*A*), the mutational effect was modest: the S_50_ for caffeine was increased by 14%, while the Hill coefficient and the high-spin content decreased by 9% and 2%, respectively (Table 1). That the n_H_ becomes lower than unity suggests a switch from noncooperative one-site to negatively cooperative multisite ligand binding. Thus, removal of the bulky side chain in R212 creates space for additional caffeines, whose association is less favorable compared to the first ligand. As expected, the loss of stabilizing contacts mediated by the guanidine group leads to a decrease in the binding affinity of the first caffeine molecule and the high-spin content. However, the small magnitude of the mutational effect suggests that the interactions mediated by R212 facilitate but are not critical for the binding of caffeine to the active site.

### Role of the intra-channel T224

**Figure 6:**
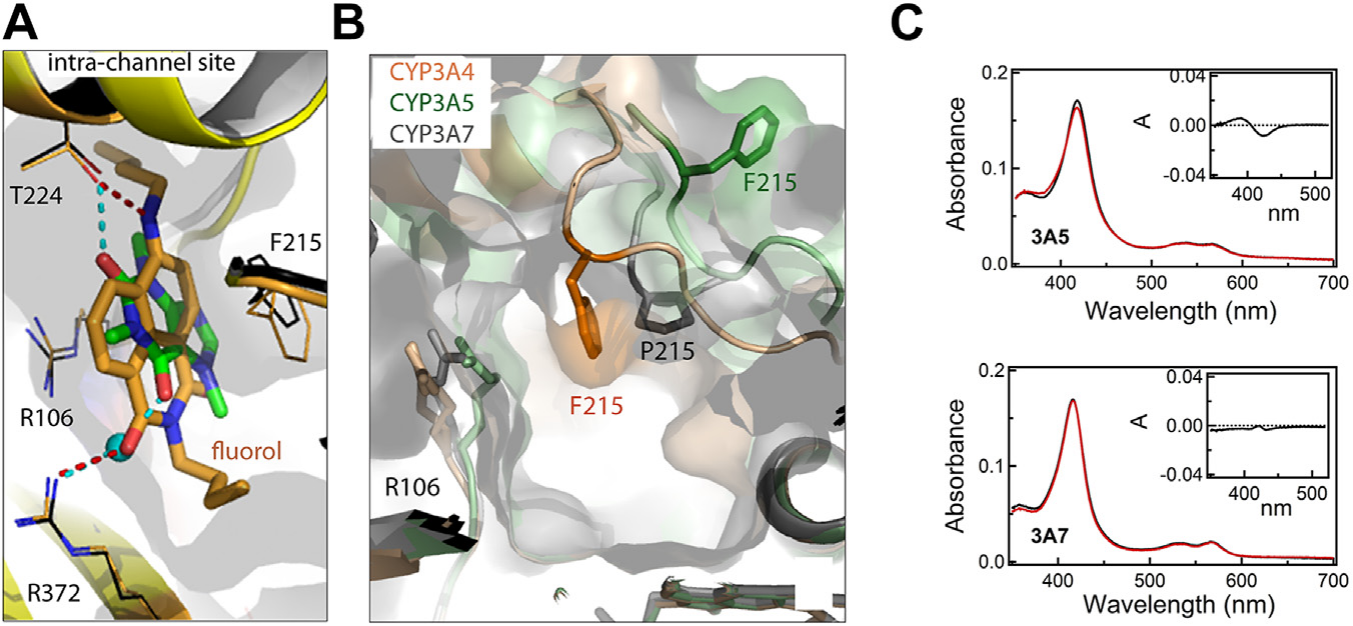
**Distinct caffeine binding ability of CYP3A enzymes.***A*, superposition of the ternary complex with caffeine (*green*/*black*) and fluorol-bound CYP3A4 (8DYC; *yellow*/*orange*). In the substrate channel, both ligands similarly stack between the R106 and F215 side chains and form H-bonds with T224 and R372. *B*, relative positioning of the channel residues R106 and F/P215 in ligand-free CYP3A4 (5VCC; *beige*/*orange*), CYP3A5 (6MJM; *green*) and CYP3A7 (7MK8; *gray*/*black*). *C*, absorbance spectra of CYP3A5 and CYP3A7 (*top* and *bottom panels*, respectively) in the absence and presence of 22 mM caffeine (in *black* and *red*, respectively). Insets are the difference spectra showing negligible perturbations in the Soret band (the *y*-axes are scaled as in insets *a* in Fig. 5). CYP3A4, cytochrome P450 3A4

Ligand association to the substrate channel was first observed in the CYP3A4-fluorol complex (23). Fluorol is a small substrate that has two binding areas in CYP3A4: the high-affinity site in the substrate channel and the low-affinity site inside the catalytic cavity. In the crystal structure (Protein Data Bank [PDB] ID 8DYC), fluorol binds to the substrate channel and occupies the same spot as caffeine, forming similar contacts with F215 and R106 and H-bonds with T224 and R372 (Fig. 6*A*). Again, aromatic stacking with fluorol is optimized through a rotational adjustment in the F215 side chain. Despite distinct incline angles of the caffeine and fluorol rings, the similarity in their positioning is remarkable.

T224 resides at the entrance of the substrate channel and its substitution with alanine eliminates the high-affinity binding site for fluorol (23). This prompted us to investigate whether the T224A mutation alters the binding of caffeine. As was observed for the WT CYP3A4, two spectral phases could be distinguished during titration of the T224A mutant with caffeine (Fig. 5*B*). However, relative to WT, the S_50_ increased by ∼4-fold and the Hill coefficient decreased by half, with no significant changes in the high-spin content (Table 1). Thus, removal of the hydroxyl group in T224 markedly lowers the binding affinity of caffeine and changes the manner of its association. The n_H_ value of 0.5 indicates that multiple caffeine molecules bind to the mutant with a pronounced negative cooperativity. One explanation for such a drastic effect could be sliding of caffeine molecules into the active site when the anchoring H-bond with T224 is no longer available. Alternatively, as noted previously (23), T224 is part of a polar network that could control the folding and mobility of the F′- and G′-helices and connecting loops. By disrupting this network, the T224A mutation could alter conformational dynamics and, consequently, the caffeine binding mode.

Notably, T224 is substituted by isoleucine in CYP3A5 and by lysine in CYP3A7. In CYP3A7, the H-bonding network involving the elongated K224 interlinks and constricts the midportion of the substrate channel (Fig. 8 in ref. (24)). Besides, CYP3A7 has a rigid proline instead of F215. In CYP3A5, F215 is conserved but points in the opposite direction, outward from the channel (Fig. 6*B*). These structural distinctions could disfavor the binding of caffeine inside the channel. Indeed, although the active site in CYP3A5 and CYP3A7 is spacious enough to accommodate at least two caffeine ligands, only negligible spectral perturbations were observed in both isoforms when a large excess of caffeine was present (Fig. 6*C*). Collectively, these findings provide further support for our hypothesis that the channel architecture controls the ligand association to CYP3A enzymes and, in CYP3A4, is predisposed for the binding of small planar polyaromatic molecules bearing polar groups (23, 24).

### Interactions at the peripheral site

That the peripheral surface adjacent to the Phe-cluster can serve as a ligand-binding site was first observed in the crystal structure of CYP3A4 complexed with substrate progesterone (PRG) (PDB ID 1W0F) (14). PRG was later cocrystallized with CYP3A4 under two different conditions (5A1P and 5A1R structures) but the docking site and contacts with the surrounding residues, particularly F219 and D214, remained the same (13). Examination of crystal lattice led to a conclusion that the binding of PRG to the outer surface could be assisted by protein-protein contacts (13). Computer simulations on monomeric CYP3A4 showed that, indeed, PRG and residues at/near the peripheral site quickly reorient to stabilize the ligand orientation but contacts with F219 are preserved (25, 26).

**Figure 7:**
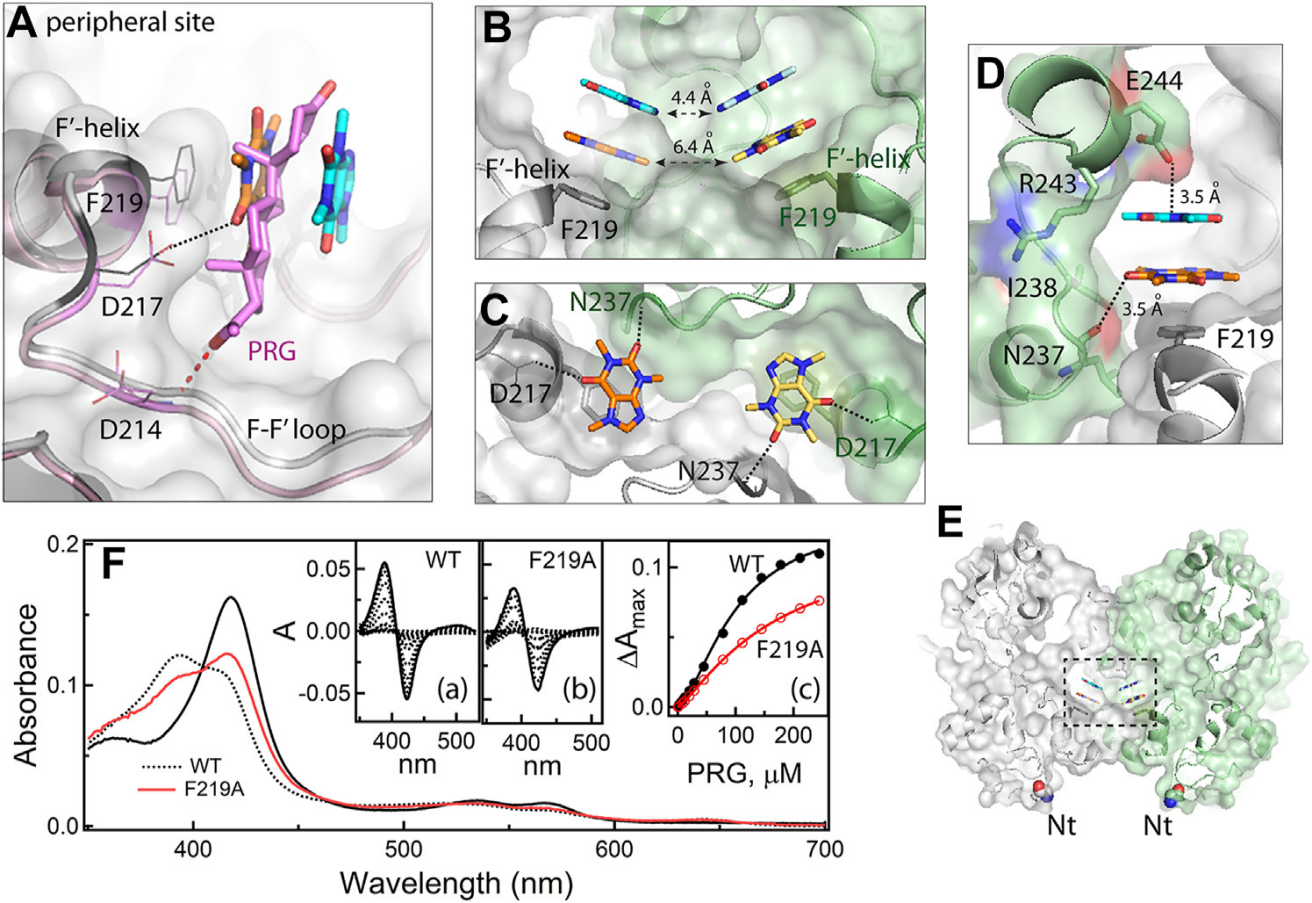
**Contacts between CYP3A4 molecules could affect the ligand binding to the peripheral site.***A*, superposition of the senary complex with caffeine and progesterone (PRG)-bound CYP3A4 (5A1R; in *pink*). At the peripheral site, PRG nearly coincides with the proximal caffein and H-bonds to the D214 carbonyl. *B*–*D*, different views at the crystallographic interface of caffeine-bound CYP3A4. Both peripheral ligands form van der Waals, aromatic or/and long-range polar contacts with residues from the symmetry related molecule (in *green*). The distal caffeine in panel *C* and symmetry related caffeines in panel *D* were removed for better presentation. *Black dotted lines* indicate attractive long-range polar interactions with the main/side chain atoms of D217 and E244 and repulsive with the N237 carbonyl group. *E*, a side view at the crystallographic dimer of CYP3A4, showing that the peripheral site lies at the interface (boxed; the N-termini are indicated). *F*, equilibrium titration of WT and F219A CYP3A4 with PRG. The main panel displays absorbance spectra recorded before (*solid black line*) and at the end of titrations (*dotted black* and *red*). Insets *a* and *b* are difference spectra recorded during titration of WT and the F219A variant, respectively. Inset *c* shows titration plots with sigmoidal fittings. The derived S_50_ and n_H_ values were 103 μM and 1.65, respectively, for WT and 158 μM and 1.25 for F219A Δ3-22 CYP3A4. CYP3A4, cytochrome P450 3A4.

When the senary complex and the PRG-bound structure are superimposed, the proximal caffeine nearly coincides with PRG (Fig. 7*A*). In the crystal lattice, the proximal caffeine is too far from the symmetry-related ligand to establish van der Waals or aromatic interactions (6.4 Å edge-to-edge distance). As in the ternary complex, one carbonyl oxygen of the proximal caffeine forms a long-range polar interaction with the D217 carboxyl group. This stabilizing contact is counterbalanced by a weak electrostatic repulsion between the second carbonyl group and the main-chain carbonyl of the symmetry related N237 (Fig. 7, *B*–*D*).

**Figure 8:**
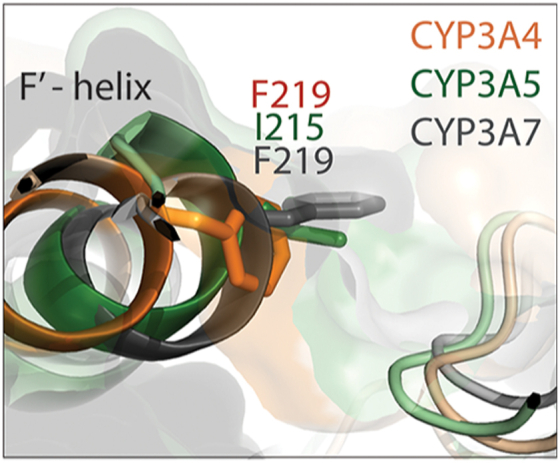
**Structural comparison of ligand-free CYP3A4 (5VCC; *orange*), CYP3A5 (6MJM; *green*) and CYP3A7 (7MK8; *gray/black*)**. Semi-transparent surfaces are displayed to show differences in the width and depth of the peripheral grove. CYP3A4, cytochrome P450 3A4.

In contrast, the distal caffeines are close enough for aromatic interactions (4.4 Å away) and flanked from top by the symmetry related E244 (3.5 Å away; Fig. 7*D*). As a result, the caffeine dimer is sandwiched between the F219 and E224 side chains, which could both promote and stabilize the caffeine binding. Thus, to some extent, association of caffeine to the peripheral site might be assisted by crystal packing or dimerization of CYP3A4. As discussed in detail previously (13), dimerization through hydrophobic F′-G′-helices can occur upon CYP3A4 isolation from membranes or incorporation into a lipid bilayer with a high protein content. Whether the crystallographic dimer resembles the naturally formed one remains to be established.

### Role of F219

F219 is a surface residue that lines the wall of the peripheral site and assists the binding of caffeine and PRG through hydrophobic and aromatic interactions (Fig. 7*A*). To elucidate how these interactions contribute to the binding affinity of both substrates, F219 was replaced with alanine. Compared to WT, the high-spin shift induced by caffeine in F219A CYP3A4 was smaller (by 8%), and the first spectral phase became negligible (Fig. 5*C*). Thus, perturbations in the heme environment depend on the composition/conformation of the peripheral site. Further, the titration plot was best fitted with a two-site rather than one-site binding equation, giving the *K*_d_ for the high- and low-affinity sites of 0.18 and 15 mM, respectively (Table 1). The percentage of the absorbance change due to the association of caffeine to the high-affinity site was only 10%. This means that the low-affinity site remains the preferable docking area, possibly because the high-affinity site is suboptimal or not fully accessible. Even so, the fact that the high-affinity site is detected only upon F219 elimination allows to speculate that (i) the peripheral surface may serve as an initial docking area with the same affinity for caffeine as the active site, owing to which the two sites cannot be spectrally distinguished in WT CYP3A4 and (ii) removal of aromatic F219 promotes caffeine association to the peripheral site, for example, by allowing the ligand to slide deeper and bind tighter to the hydrophobic grove. Alternatively, the high-affinity area might be located elsewhere and is unavailable in WT due to conformational constraints that could be eliminated by the F219A mutation.

The substitution of F219 with alanine perturbed the binding of PRG as well (Fig. 7*E*). WT CYP3A4 binds PRG with a pronounced positive cooperativity: S_50_ and n_H_ of 103 ± 3 μM and 1.65 ± 0.07, respectively. For the F219A mutant, the corresponding values were 158 ± 9 μM and 1.25 ± 0.05 (53% increase and 27% decrease, respectively), while the high-spin content dropped from 70% to 45%. Thus, F219 contributes to the binding affinity and cooperativity of PRG and controls spin transition in the heme.

F219 is conserved in CYP3A7 but corresponds to I215 in CYP3A5. Structural comparison of CYP3A enzymes shows that the peripheral grove is the widest/deepest in CYP3A4 and most shallow/narrow in CYP3A7, partly due to horizontal orientation of the F219 ring (Fig. 8). These differences are important because the F′-helix bearing F219 is predicted to directly contact or partially embed into the lipid bilayer in the natural membrane environment (27, 28). Computer simulations on membrane-bound CYP3A4 showed that the F′-G′-helix/lipid interactions define the shape of the peripheral pocket and strengthen the ligand association to this dynamic site (29, 30, 31, 32). Moreover, molecules bound to the peripheral site are thought to exert allosteric effects through modulation of the F′-G′ helix mobility which, in turn, could alter the shape/volume of the substrate-binding pocket, stabilize the oxy complex, and change the regiospecificity of metabolism of the active site substrate(s) (26, 29, 30, 31, 33, 34). Our results support this point of view and provide further evidence that the composition/architecture of the peripheral site, especially around residue 219, could modulate the ligand binding and cooperativity in CYP3A enzymes.

With regard to the interaction of caffeine with membranous CYP3A4, it should be pointed out that adsorption of caffeine monomers from an aqueous environment into a lipid bilayer is energetically favored but, upon partitioning, caffeine molecules reside on the outward aqueous side in the proximity of polar lipid headgroups (35). Caffeine stacks, on the other hand, do not spontaneously transition into membranes (36). Thus, caffeine stacking to the peripheral F219 could be physiologically important and facilitate the transport of both monomers and oligomers of caffeine into the active site of microsomal CYP3A4.

### Comparison of the senary complex and *cis*-10 bound CYP3A4

**Figure 9:**
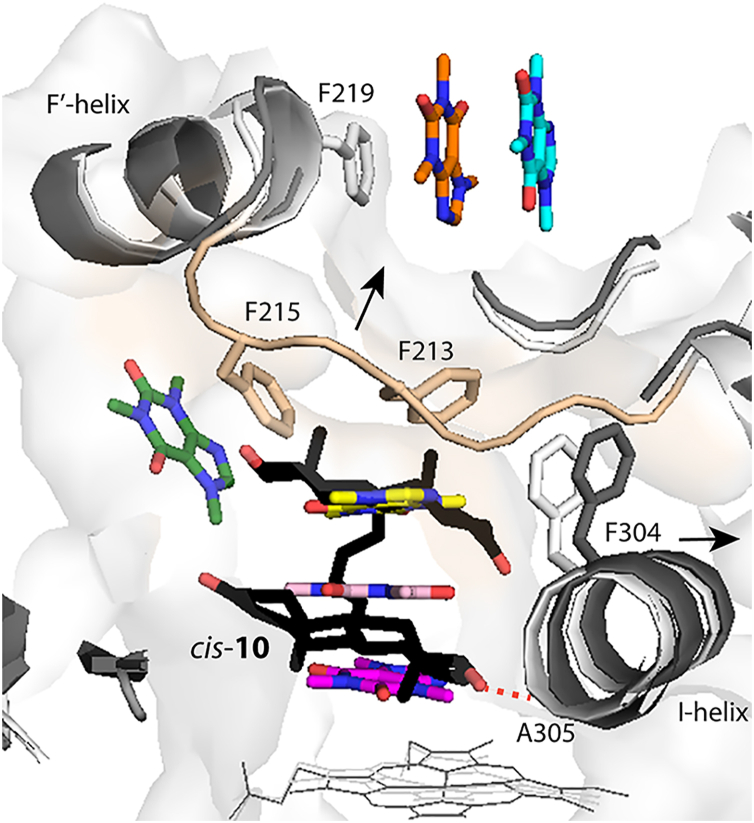
**Superposition of the senary complex (*light gray/beige*) and *cis*-10 bound CYP3A4 (*dark gray*/*black*; PDB ID****7LXL****)**. *cis*-10 (in *black sticks*) represents a testosterone dimer linked by a four-carbon, double-bond containing aliphatic chain. Sterol moieties of *cis*-10 are parallel to each other and tilted relative to the heme. The proximal sterol occupies space between two lower caffeines and, due to steric hindrance, displaces the I-helix. This sterol is in a productive orientation stabilized by an H-bond with the A305 carbonyl (*red dotted line*). The distal sterol coincides with the upper caffeine and clashes with the F-F′-loop (in *beige*), leading to its disorder. Expansion of the active site needed for the *cis*-10 binding is indicated by *arrows*. CYP3A4, cytochrome P450 3A4; PDB, Protein Data Bank.

Until now, there were no structures of CYP3A4 bound to multiple substrates. However, the complex with *cis*-10, a testosterone dimer linked by a four-carbon, double-bond containing aliphatic chain (PDB ID 7LXL), sheds light on how two bulky sterol molecules could fit into the active site (37). Structural superposition shows (Fig. 9) that, overall, the senary complex and *cis*-10 bound CYP3A4 are similar, with the r.m.s. deviation between the C_α_ atoms less than 0.55 Å. One distinction is a tilted rather than parallel orientation of *cis*-10 relative to the heme, which allows to minimize steric clashing with the surrounding residues. Still, due to steric hindrance, the I-helix becomes distorted, and the F-F′-loop completely disordered. Notably, the distal sterol coincides with the upper caffeine ligand, whereas the proximal sterol occupies space between two lower caffeines and is fixed in a productive orientation through an H-bond with the A305 carbonyl. Hydrogen bonding is thought to play an important role in CYP-substrate interactions by contributing to the overall binding affinity and assisting the substrate recognition and orientation in the active site (38). The caffeine and *cis*-10 bound structures support this notion and show how residues in the vicinity of the heme (R105, S119, R212, A305, and R372) can contribute to the formation/stabilization of productive complexes with substrates. Moreover, the structures demonstrate that the active site of CYP3A4 is spacious enough to accommodate several small molecules without drastic conformational changes but can easily expand to allow binding of larger substrates.

## Conclusions

This study was carried out to gain structural information on the interaction of CYP3A4 with caffeine, a commonly consumed psychoactive alkaloid serving as a substrate and modulator of CYP3A4. Determination of crystal structures of CYP3A4 complexed with three and six molecules of caffeine provided the first direct insights into multiple substrate binding.

Depending on the availability of caffeine, the active site could accommodate one or a stack of three caffeines, with no conformational adjustments needed except reorientation of the R212. Caffeins proximal to the heme orient suitably for C8-hydroxylation or 3-N-demethylation, catalyzed by CYP3A4 *in vivo*. Nonetheless, the senary complex is likely to be nonfunctional because, by clogging the active site, the caffeine trimer could preclude product dissociation and multiple turnovers.

The study emphasized the importance of remote ligand-binding sites and identified the intra-channel T224 and peripheral F219 as key residues promoting caffeine association. Interaction with F219 was found to contribute to the binding affinity and cooperativity of PRG as well, raising the possibility that this residue could play a more prominent role and mediate the association and allosteric effects of other ligands.

The study provided further evidence that the substrate channel in CYP3A4 is predisposed for the binding of planar polyaromatic molecules containing polar groups. Moreover, it allowed to conclude that the channel architecture is an important factor contributing to the distinct ligand binding ability of CYP3A enzymes.

The CYP3A4-substrate complexes crystallized so far are scarce but very important for understanding and prediction of xenobiotic binding and metabolism. The new structural and experimental data expand the knowledge on structure-function of CYP3A4 and could help improve the outcomes for in silico modeling of the binding modes, metabolic stability, and inhibitory potential of purine-based drugs and other small aromatic compounds.

## Experimental procedures

### Protein expression and purification

Human full-length and Δ3-22 CYP3A4, as well as the R212A and T224A variants were produced as reported previously (21, 39). The F219A mutation was introduced using PrimeSTAR Max DNA Polymerase master mix (Takara Bio) and confirmed by sequencing. The F219A variant of Δ3-22 CYP3A4 was expressed and purified using the protocol developed for WT CYP3A4 (39). All proteins used in the study had R_z_ (A_416/280 nm_ ratio) of 1.5 or higher.

### Spectral measurements

Equilibrium ligand binding to CYP3A4 was monitored on Cary 300 spectrophotometer at 23 °C in 0.1 M potassium phosphate, pH 7.4, supplemented with 10% glycerol and 1 mM DTT. A 200 mM stock of caffeine (Sigma-Aldrich) was prepared in hot water and then diluted with ambient water to make 5 to 100 mM working solutions, which were added to the 1.5 μM protein solution in small aliquots. Before recording absorbance spectra, the mixture was allowed to equilibrate for 10 to 15 min. The difference spectra were obtained by subtracting absorbance spectra recorded after each caffeine addition from the reference spectrum of ligand-free CYP3A4.

For experiments with PRG, a 50 mM stock was made in dimethyl sulfoxide and diluted with the same solvent to prepare 1 to 10 mM working solutions. During titrations, small aliquots of PRG were added to the experimental cuvette, while the same amount of solvent was added to the reference cuvette that contained a similar protein solution. The difference absorbance spectra were recorded 10 to 15 min after addition of PRG, when no further spectral perturbations could be detected. The recorded spectra were corrected for dilution. Absorbance changes (peak-to-trough difference) were plotted *versus* ligand concentration and fitted using one- or two-site binding model to derive the dissociation constant(s), *K*_d_. Sigmoidal plots were fitted using the Hill equation to derive S_50_ and n_H_. The binding parameters are given in Table 1 or the main text.

The high spin content in substrate-bound CYP3A4 was estimated based on the absorbance spectra of the ligand-free protein (considered as 100% low-spin) and the bromocryptine-bound form (100% high-spin conversion).

### Crystallization of the CYP3A4-caffeine complexes

WT Δ3-22 CYP3A4 (100 mg/ml) in 0.1 M potassium phosphate pH 7.4, supplemented with 20% glycerol, 2 mM DTT and 0.1 M NaCl was mixed with an aqueous solution of 100 mM or 250 mM caffeine to achieve the final ligand concentration of 22 mM and 80 mM, respectively. After 20 min incubation, the mixture was centrifuged to remove the precipitate. The supernatant (0.5 μl) was combined with an equal volume of 10% PEG 3350, containing 3% tacsimate pH 8.0 (Hampton Research), and equilibrated against the same precipitant solution using a sitting drop vapor diffusion setup. Crystals grew within several days and, upon harvesting, were cryoprotected with Paratone-N oil and frozen in liquid nitrogen.

### Determination of the X-ray structures

X-ray diffraction data were collected at the Stanford Synchrotron Radiation Lightsource beamline 9 to 2. The high resolution cutoffs were based on the Pearson correlation coefficient (CC1/2 ≥ 0.3) (40) and signal-to-noise ratio (*I*/σ*I* ≥ 1.0). Crystal structures were solved by molecular replacement with PHASER (41) and 5VCC structure as a search model. The ligand was built with eLBOW (42) and manually fit into the density with COOT (43). The initial models were rebuilt and refined with COOT (https://www.ucl.ac.uk/∼rmhasek/coot.html) and PHENIX (https://phenix-online.org/) (42). Polder omit electron density maps were calculated with PHENIX. The final orientation of caffeine molecules was chosen based on the best fit into electron density and the lowest R/R_free_-factors of the refined structures. Data collection and refinement statistics are summarized in Table 2. The atomic coordinates and structure factors for CYP3A4 complexed with three and six caffeine molecules were deposited to the PDB with the ID codes 8SO1 and 8SO2, respectively.

## Data availability

All experimental data generated during this study are included in this article and the accompanying supporting information file. Coordinates and structure factors for the X-ray models of caffeine bound CYP3A4 are publicly available at the Protein Data Bank (https://www.rcsb.org/).

## Supporting information

This article contains supporting information.

## Conflict of interest

The author declares no conflict of interest with the contents of this article.
