# Supplementary material for: Interaction of CYP3A4 with caffeine: First insights into multiple substrate binding
Source: J Biol Chem. 2023 Jul 29;299(9):105117. doi: 10.1016/j.jbc.2023.105117 (PMC10470200; doi:10.1016/j.jbc.2023.105117)
Supplement: Supporting information [file mmc1.pdf]

SUPPORTING INFORMATION

Interaction of CYP3A4 with caffeine: First insights into multiple substrate binding

Irina F. Sevrioukova

Department of Molecular Biology and Biochemistry, University of California, Irvine, California 92697-3900.

Content: page

Figure S1 - Spectral changes observed during titration of full-length CYP3A4 with caffeine S2

Figure S2 - Polder omit electron density maps for individual caffeine ligands S3

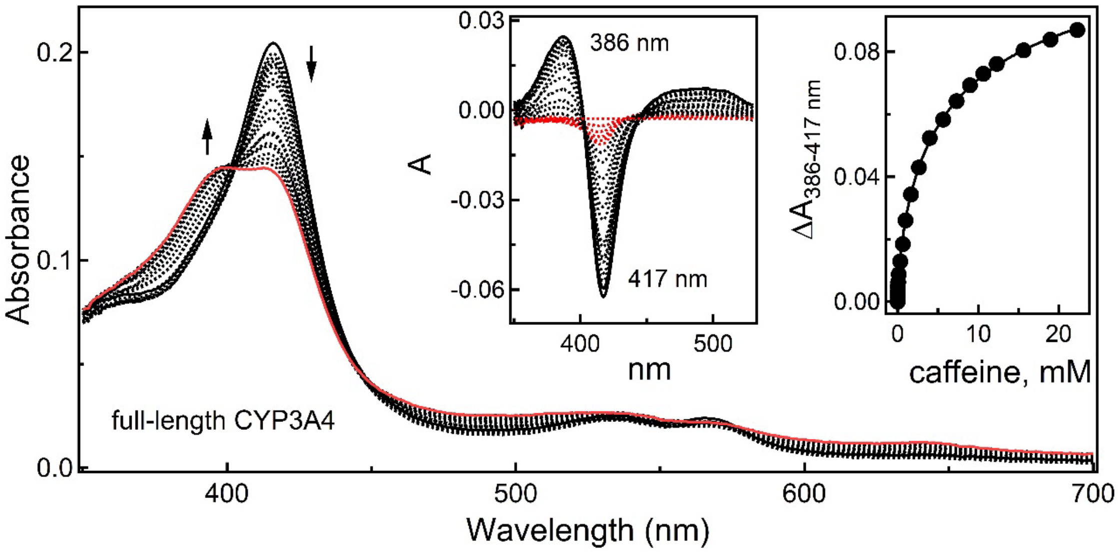

Figure S1. Spectral changes observed during equilibrium titration of full-length CYP3A4 with caffeine. In the main panel, arrows indicate direction of absorbance changes. The red spectrum was recorded at the end of the experiment. Difference spectra are shown in inset *a*. Absorbance changes taking place during the first spectral phase, characterized by a decrease in the Soret band with no rise in the 350-400 nm region, are highlighted in red. Inset *b* is a titration curve that was best fit to a 2-site binding model (solid line). The derived dissociation constants for the high- and low-affinity sites were 20.5 ± 0.6 μM and 4.5 ± 0.3 mM, respectively. The percentage of the absorbance change due to association of caffeine to the high- and low-affinity sites was 9% and 91%, respectively. The high-spin content in the caffeine-bound form was 44 ± 2%.

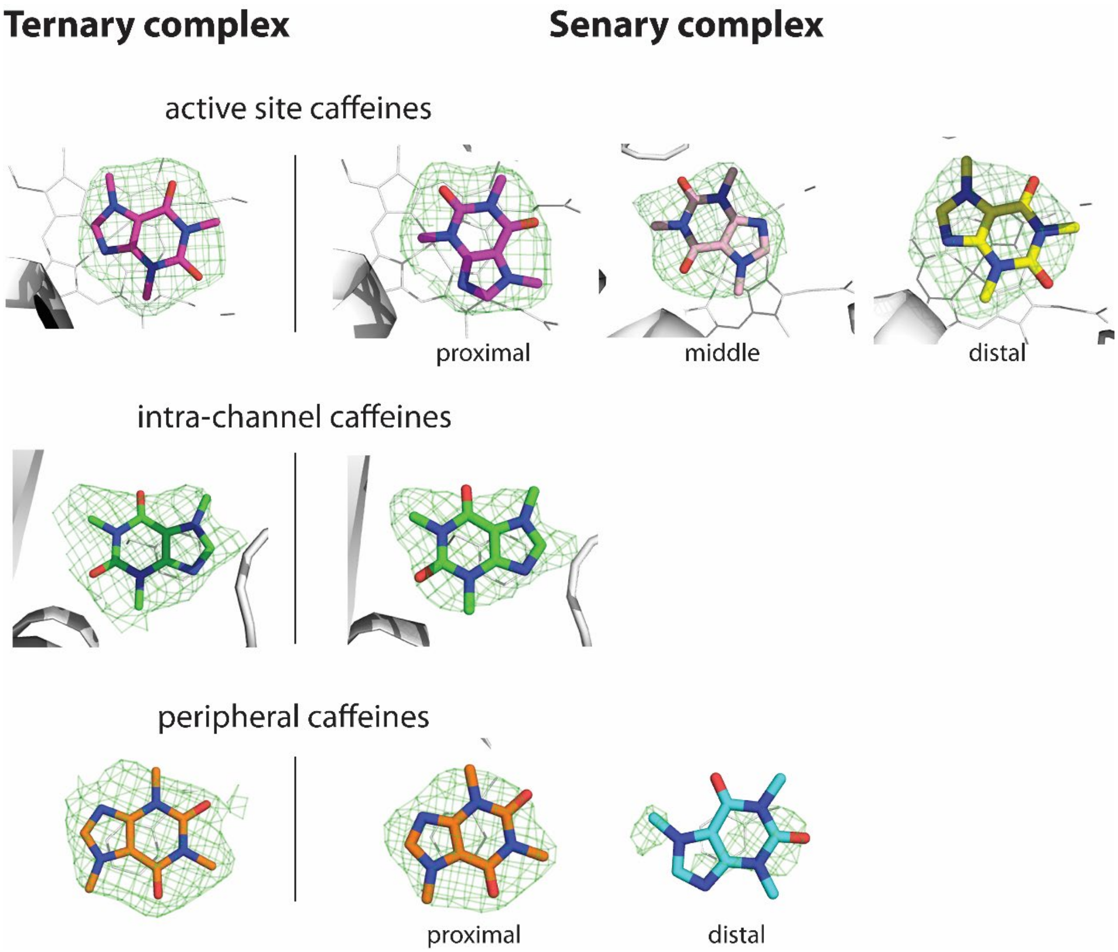

Figure S2. Polder omit electron density maps for caffeine ligands in the ternary and senary complexes (green mesh; contoured at 3σ level). The orientation of caffeine molecules was chosen based on the best fit into electron density and the lowest R/R_{free}-factors of the refined structures (measure of the agreement between the model and the experimental X-ray diffraction data).
